# Morphological Characteristics of Biliary Strictures after Liver Transplantation Visualized Using SpyGlass™ Cholangioscopy

**DOI:** 10.1155/2020/8850000

**Published:** 2020-08-03

**Authors:** Yathip M. Chokpapone, Anne R. Murray, Ashwini P. Mehta, Vichin C. Puri, Alejandro Mejia, Parvez Mantry

**Affiliations:** ^1^The University of Texas at Dallas, Dallas, 800 W Campbell Rd., Richardson, TX 75080, USA; ^2^Clinical Research Institute, Methodist Health System, 1411 N. Beckley Ave, Pavilion III, Suite 168, Dallas, TX 75203, USA; ^3^The Liver Institute, Methodist Dallas Medical Center, 1411 N. Beckley Ave, Pavilion III, Suite 268, Dallas, TX 75203, USA

## Abstract

Biliary complications following liver transplant are common. Endoscopic retrograde cholangiopancreatography (ERCP) and magnetic resonance cholangiopancreatography (MRCP) are the main techniques used to diagnose and treat biliary complications; however, these techniques have limits to the depth of visualization. In this report, we present five cases of orthotopic liver transplant patients with biliary complications that underwent ERCP- or MRCP-guided cholangioscopy with the SpyGlass™ DS Direct Visualization System (SDDVS). The SDDVS allowed for the visualization of the morphological characteristics of biliary strictures, and images collected using the SDDVS allowed for four of the cases to be treated endoscopically. Our findings suggest that cholangioscopy with the SDDVS is a promising method to guide the endoscopic treatment of biliary complications after liver transplantation.

## 1. Introduction

Biliary complications such as biliary leakage, biliary stones, or bile duct (anastomotic or nonanastomotic) strictures affect 10 to 25% of adult liver transplant recipients [1–5]. These complications often lead to increased morbidity as well as reduced graft and patient survival [6]. Currently, endoscopic retrograde cholangiopancreatography (ERCP) and magnetic resonance cholangiopancreatography (MRCP) are the main techniques used to diagnose and treat biliary complications in liver transplant patients [7]. However, due to limits to the depth of visualization in these techniques, up to 30% of biliary strictures are unable to be characterized [8].

The single-operator SpyGlass™ Direct Visualization System (Boston Scientific) increased bile duct clarity and improved image quality, resulting in reduced surgical complication rates [9, 10]. The improved image aspect in the SpyGlass™ Direct Visualization System allowed endoscopists to perform several procedures, including the evaluation of biliary strictures, examination of suspected benign and malignant conditions, passage of a guide wire in difficult strictures, and the removal of large stones or foreign objects from the common bile duct (CBD) [10, 11]. Later, the SpyGlass™ DS Direct Visualization System (SDDVS) further improved image resolution, improved ergonomics and stability, and simplified ERCP-guided cholangioscopies by incorporating a fully integrated catheter and a single-use scope [12].

Since implementation of the SDDVS, our center has observed notable morphological characteristics of biliary strictures in five postorthotopic liver transplantation (OLTx) patients.

## 2. Case Presentations

Five patients with post-OLTx bile duct strictures underwent an ERCP-guided cholangioscopy with the SDDVS at our center (Table 1). Their median age was 61.6 years (interquartile range (IQR), 49–71 years), three were males, and two were females.

### 2.1. Case I

**Figure 1:**
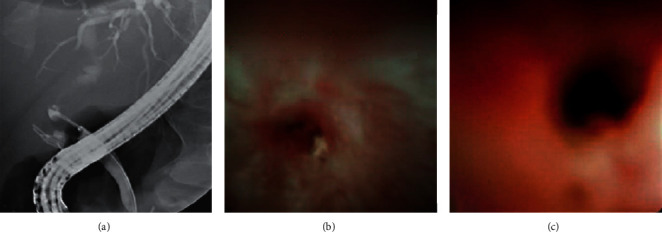
Pinhole inflammatory fibrotic biliary stricture observed using fluoroscopy (a) and the SpyGlass™ DS Visualization System (b,c).

A 71-year-old Caucasian male presented in 2017 and was diagnosed with cryptogenic end-stage liver disease (ESLD) complicated by portal vein thrombosis and hepatic hydrothorax. He had an OLTx on November 19, 2018, and received a cytomegalovirus (CMV) total antibody-positive and Epstein–Barr virus (EBV) viral capsid antigen (VCA) IgG-positive deceased donor organ. His postoperative course was unremarkable, and he had no acute rejection or infections. He underwent a MRCP-guided cholangioscopy for elevated liver function tests (LFTs) on October 23, 2019, which showed both intra- and extrahepatic biliary dilatation. He subsequently underwent an ERCP-guided cholangioscopy with the SDDVS on October 31, 2019. The procedure showed a nonnegotiable (with a 0.021 inch wire) pinhole inflammatory fibrotic stricture of the bile duct as well as anastomosis with excess exudate that was not amenable to stenting (Figure 1). The patient eventually underwent a Roux-en-Y choledochojejunostomy that resolved the stricture and is doing well. The SDDVS was useful for defining stricture morphology, attempting wire cannulation, and for helping the endoscopist make a prompt decision to pursue surgical treatment of the stricture.

### 2.2. Case II

**Figure 2:**
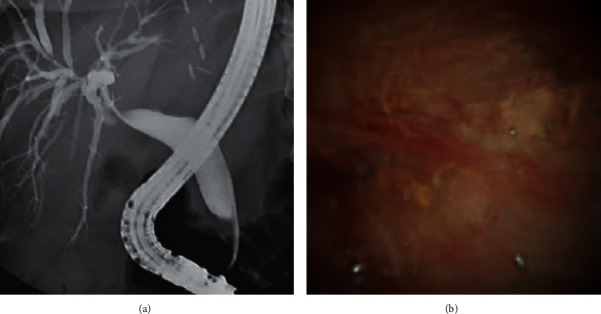
Severely tight anastomotic, fibrotic stricture and a pinhole orifice stricture observed using fluoroscopy (a) and the SpyGlass™ DS Visualization System (b).

A 62-year-old Hispanic female presented in 2014 and was diagnosed with cryptogenic cirrhosis complicated by portosystemic encephalopathy, anasarca, ascites, portal hypertensive gastropathy, and portal vein thrombosis. She had an OLTx on November 19, 2018, and received a CMV total antibody-positive deceased donor organ. Her postoperative course was complicated by herpes simplex pneumonia and candidemia, but she did not experience any episodes of acute rejection. She underwent an ERCP-guided cholangioscopy for abnormal LFTs on January 14, 2019, which showed a tortuous, redundant CBD that was difficult to cannulate. A biliary sphincterotomy was performed, and a 5 FR 10 cm straight plastic stent was placed across the CBD; however, the wire could not negotiate the stricture. She proceeded to have four additional ERCP-guided cholangioscopies with four stent placements throughout 2019. On November 14, 2019, she had an ERCP-guided cholangioscopy with the SDDVS. The SDDVS revealed a severely tight anastomotic, fibrotic stricture and a pinhole orifice (Figure 2). The SDDVS helped us assess stricture morphology, enabled multiple endoscopic metal stent replacements, and permitted resolution of the stricture, thereby avoiding surgery. The patient is currently doing well and has not required another ERCP or stent placement.

### 2.3. Case III

**Figure 3:**
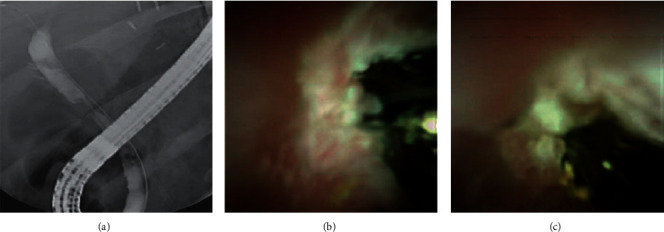
Anastomotic bile stricture with persistent scarring observed using fluoroscopy (a) and the SpyGlass™ DS Visualization System (b,c).

A 64-year-old Caucasian male presented in 2017 and was diagnosed with autoimmune hepatitis cirrhosis. He underwent an OLTx on April 05, 2019, and received a CMV total antibody-positive deceased donor organ. He had a complicated postoperative course that involved acute cellular rejection (rejection activity index (RAI) = 3), which initially improved with an increased dosage of Prograf®. He was subsequently readmitted for fever and elevated LFTs. He underwent a liver biopsy on April 11, 2019, that revealed acute cellular rejection (RAI = 3–4) and was treated with steroids. The patient had an ERCP-guided cholangioscopy on April 29, 2019 for elevated LFTs, which showed anastomotic stenosis and a low-grade leak. A biliary sphincterotomy was performed, and a 10 FR 9 cm plastic straight bile duct stent was placed across the leak. He had four additional ERCPs with stent placements throughout 2019. On November 14, 2019, he had an ERCP-guided cholangioscopy with the SDDVS that revealed an anastomotic bile duct stricture with persistent scarring (Figure 3). A CBD stone and debris were evacuated, and a metal stent was replaced that resolved the stricture. The patient is currently doing well and has not needed another ERCP-guided cholangioscopy or stent placement.

### 2.4. Case IV

**Figure 4:**
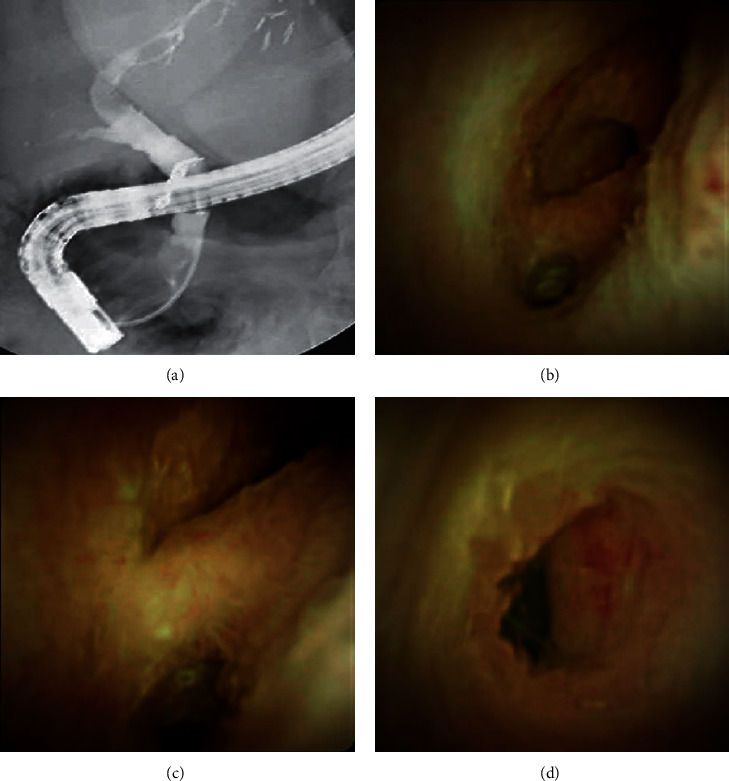
Scarring in the common bile duct located using fluoroscopy (a) and the SpyGlass™ DS Visualization System (b–d).

A 49-year-old Caucasian female presented in 2017 and was diagnosed with ESLD from primary sclerosing cholangitis complicated by hepatic decompensation from ascites, edema, and hepatocellular carcinoma (HCC). She underwent an OLTx on June 27, 2018, and received a CMV total antibody-positive and EBV VCA IgG-positive deceased donor organ. The patient had a notable postoperative course due to an episode of severe rejection (RAI = 9; resolved with RAI = 3) with both cellular- and antibody-mediated components. She had an ERCP-guided cholangioscopy performed on November 06, 2018, for abnormal LFTs that showed a deeply cannulated CBD, a tight anastomotic biliary stricture, and a somewhat prominent proximal biliary tree. A biliary sphincterotomy was performed, and a 4×4 balloon dilator was used to dilate the stricture. While a 10 FR 10 cm stent was not able to pass across the stricture, a 7 FR 10 cm straight plastic stent passed across the stricture. She had four additional ERCP-guided cholangioscopies with two stent placements. On September 10, 2019, she had an ERCP-guided cholangioscopy with the SDDVS. The SDDVS showed scarring in the CBD, but no narrowing; the ostia of the hepatic ducts appeared to be normal (Figure 4). The metal stent, a CBD stone, and debris were removed, and the biliary anastomotic stricture was resolved. Scar tissue remained in the CBD, but no internal strictures were seen. She is currently doing well without any complaints and has not required another ERCP-guided cholangioscopy or stent placement.

### 2.5. Case V

**Figure 5:**
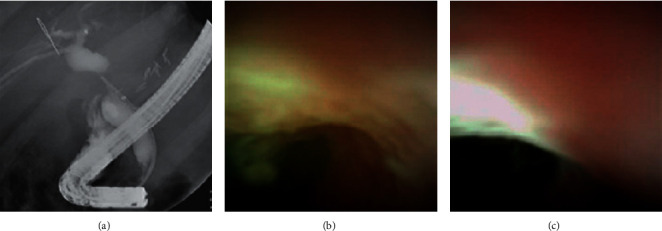
Scarring in the anastomosis observed using fluoroscopy (a) and the SpyGlass™ DS Visualization System (b,c).

A 62-year-old African American male presented in 2017 and was diagnosed with ESLD from hepatitis C virus that was complicated by HCC. He achieved sustained virologic response and had a microwave ablation in July 2015 for the HCC. He had an OLTx on May 22, 2017, and received a CMV total antibody-positive and EBV VCA IgG-positive deceased donor organ. He had a prolonged postoperative course due to acute cellular rejection (RAI = 7; resolved with RAI = 3), antibody-mediated rejection, and a nonhealing wound from the operation that required a wound vac. He had an ERCP-guided cholangioscopy on August 01, 2017, after an abnormal MRCP-guided cholangioscopy. The ERCP showed an anastomotic biliary stricture and common hepatic duct stricture. A 10 FR 12 cm plastic straight bile duct stent was placed. He had two subsequent ERCP-guided cholangioscopies and one bile duct stent placed. On August 28, 2018, he underwent an ERCP-guided cholangioscopy with the SDDVS that showed a web-like stricture at the anastomosis. The SDDVS permitted the negotiation of a wire through the stricture, and the stricture was dilated with a 4×4 hurricane balloon dilator. A 10 FR 10 cm straight plastic stent was placed across the CBD. Following this, he had four more ERCP-guided cholangioscopies with four stent placements. On November 14, 2019, he underwent an ERCP-guided cholangioscopy with the SDDVS that showed scarring in the anastomosis (Figure 5). A healthier mucosa in the proximal CBD was seen using the SDDVS, and the metal stent was replaced in addition to CBD stones and debris removed.

## 3. Discussion

The image quality of the SDDVS allows endoscopists to distinguish between anastomotic and nonanastomotic strictures because they are able to observe epithelial changes, ulcers, small stones, and bile casts that they are unable to detect by ERCP [13]. However, the system has several limitations (e.g., the high cost of the system; the small working channel diameter that does not allow for the use of some devices; a learning curve in the training of endoscopists to use this system and techniques; and a lack of guidelines for its use) that limit the widespread use of the SDDVS [11, 14, 15].

Only a few studies have used the earlier generation of the SpyGlass™ system for the management of biliary complications after liver transplant [16–21]. The use of the newer generation SDDVS in the management of liver transplant-associated biliary complications has not been reported [5]. We have demonstrated the successful use of ERCP-guided cholangioscopy using SDDVS in characterizing anastomotic biliary strictures to guide endotherapy post-OLTx and that SDDVS may help with prognostication. In our case series, all except one patient was able to be treated endoscopically. Using the SpyGlass™ system, we were also able to discover that the strictures in these patients were heavily fibrotic in nature. Our findings may guide future novel endoscopic techniques (e.g., intraluminal needle-knife) when the technology becomes available.

SpyGlass™ cholangioscopy can distinguish a fibrotic stricture from a kink by its morphological appearance. Kinking of the CBD after OLTx is a common problem. This is likely due to a long redundant donor duct during harvesting, which is often done to prevent tension at the anastomosis. Kinking of the CBD can often mimic a stricture and sometimes poses challenges to the endoscopist during ERCP. Often, deep access to the CBD can be difficult in these patients, as the wire tends to buckle near the area of the kink. A technique used to straighten out the kink is to hyperinflate a balloon and pull it near the distal CBD. This often will straighten out the kink and enable to wire to pass into the hilar area. In terms of therapeutics, plastic stents will often resolve a kink in the CBD and can be removed at subsequent procedures 10 to 12 weeks later. In contrast, a fibrotic stricture almost always requires multiple rounds of stenting.

The fibrotic nature of the strictures provides a good rationale for the use of self-expandable metal stents to maintain long-term decompression of the strictures. Fibrotic biliary strictures are putatively the reason that OLTx patients often need 6 to 12 months of endoscopic therapy, repeated balloon dilations, and stent exchanges for stricture resolution. Endotherapy, even with a frequent need to repeat procedures every 10 to 12 weeks, is preferred at most centers compared to surgical alternatives (e.g., Roux-en-Y choledochojejunostomy) due to the surgery-associated risk of morbidity and complications. Our experience at a large tertiary center shows that approximately 90% of anastomotic biliary strictures can be successfully managed with endoscopic therapy, avoiding the need for surgery.

The limitations of our case series include its retrospective nature and small sample of patients. However, the successful use of the SDDVS is well illustrated. Continued observation using the SDDVS may be beneficial for the development of guidelines and improvement in the treatment of biliary strictures and scarring in future studies. Overall, our findings suggest that cholangioscopy with the SDDVS represents a new frontier guiding the endoscopic treatment of biliary complications after OLTx.

## Figures and Tables

**Table 1:** Patient demographics and stricture morphological characteristics.

Case no.	Age (yr)	Sex	Ethnicity	BMI	Number of days after transplant to initial diagnosis	Morphological characteristics found using the SDDVS
1	71	M	Caucasian	23	384	Pinhole inflammatory fibrotic bile duct stricture; anastomosis with excess exudate; not amenable to stenting
2	62	F	Hispanic	25	56	Severely tight anastomotic stricture; pinhole orifice; amenable to stenting
3	64	M	Caucasian	31	25	Anastomotic bile duct stricture with persistent scarring
4	49	F	Caucasian	46	132	Scarring in the common bile duct but no narrowing; ostia of the hepatic ducts appeared to be normal
5	62	M	African American	30	71	August 28, 2018: web-like stricture at the anastomosis
						November 14, 2019: scarring in the anastomosis with a healthier mucosa in the proximal common bile duct

SDDVS, SpyGlass™ DS Direct Visualization System. BMI, body mass index.

## Data Availability

Access to data is restricted to retain patient privacy. This is a novel study based on limited real-world data gathered from patients within our hospital system, so there is currently no additional supporting data in this regard.
